# Supplementary material for: KDEL receptor regulates secretion by lysosome relocation- and autophagy-dependent modulation of lipid-droplet turnover
Source: Nat Commun. 2019 Feb 13;10:735. doi: 10.1038/s41467-019-08501-w (PMC6374470; doi:10.1038/s41467-019-08501-w)
Supplement: Supplementary file 6 — Supplementary Information [file 41467_2019_8501_MOESM6_ESM.pdf]

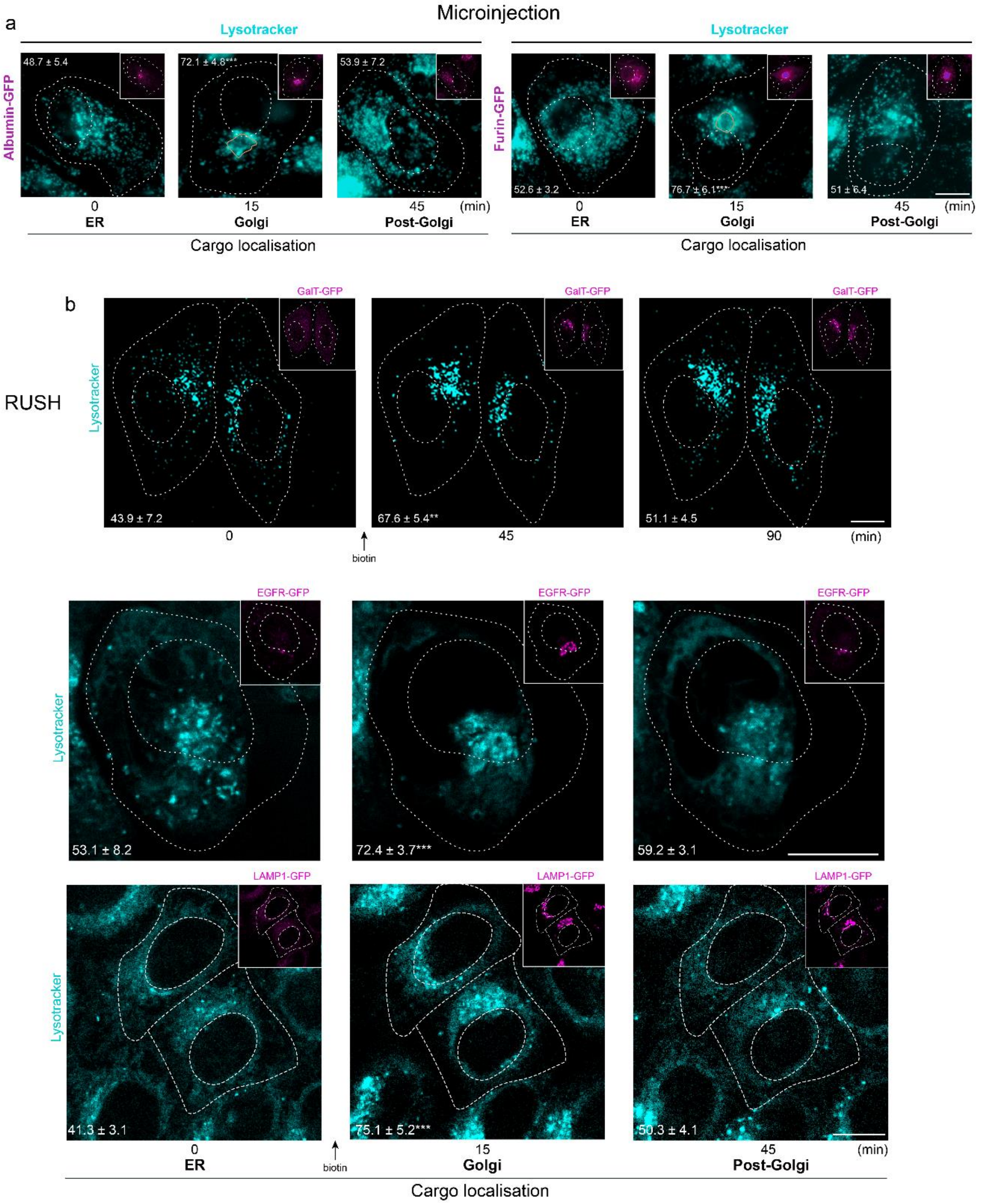

Supplementary Figure 1. Lysosome repositioning is independent of cargo type or ER to Golgi transport synchronization method.

(a) Microinjection was used to express and synchronise ER to Golgi transport of GFP-tagged version of luminal (Albumin) and transmembrane (Furin) cargo proteins on HeLa cells. After microinjection the cells were kept for 1 hour at 37°C for protein expression (ER) and then were fixed after 30 minutes (Golgi) and 60 minutes (Post-Golgi) (see inset). Radial integrated fluorescence intensity was used to calculate the perinuclear lysosome percentage localization was calculated for each selected time point (n=30 cells) and represents ER, Golgi and post-Golgi cargo localisation (inset). (b) RUSH system was used to control the ER to Golgi transport of GFP-tagged: lysosome (lysosomal-associated membrane protein 1, LAMP1), plasma membrane (Epidermal Growth Factor Receptor, EGFR) and Golgi (Galactosyltransferase, GalT) localized proteins. Cells were followed by live cell imaging before and after biotin addition to release ER block. The perinuclear lysosome percentage localization was calculated for each selected time point (n=30 cells) and represents ER, Golgi and post-Golgi cargo localisation (inset). Scale bar 10 µm. *** p <0.001 (Student's t-test).

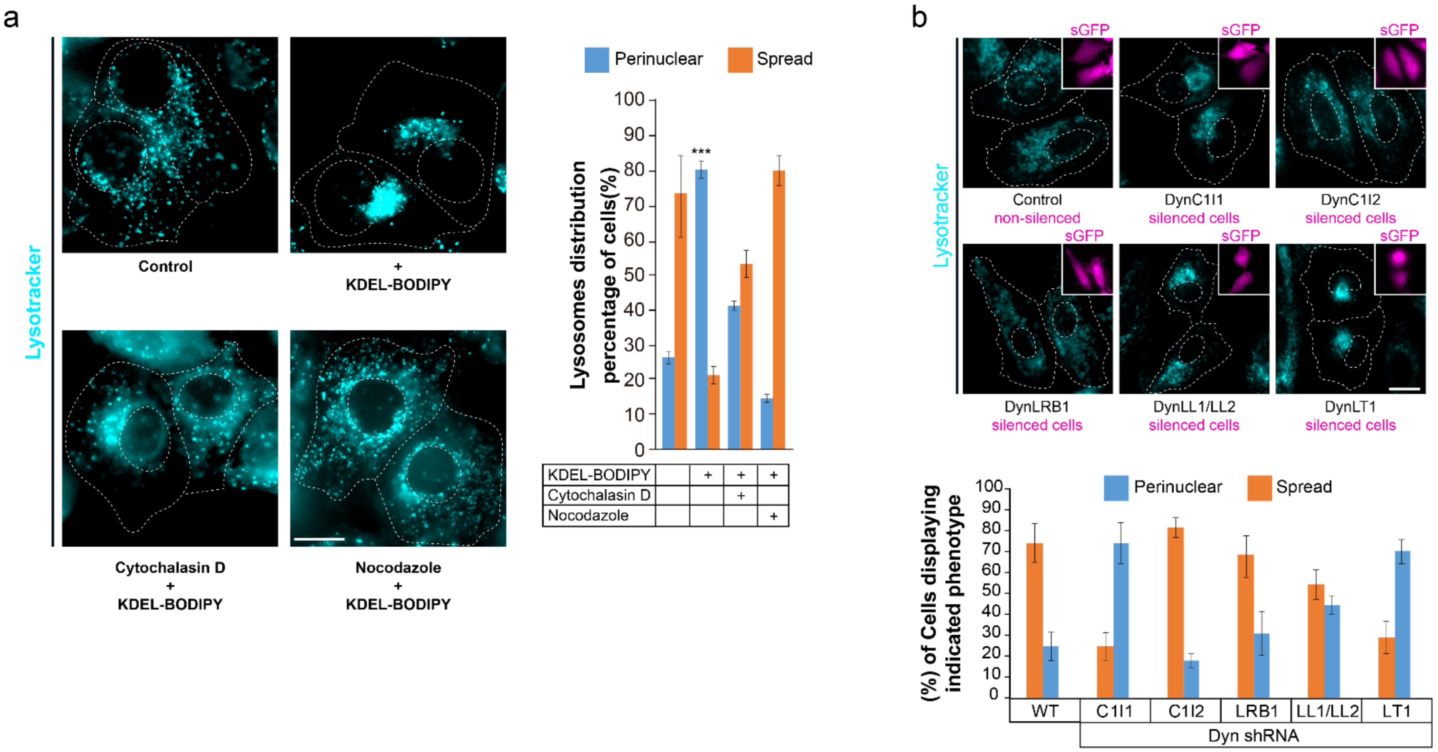

Supplementary Figure 2. Lysosome repositioning required microtubules cytoskeleton and specific dynein chain.

(a) HeLa cells were incubated with the KDELR activator KDEL-BODIPY peptide (1 µM) in the absence or presence of actin (cytochalasin D, 1 µM) or microtubules (nocodazole, 33 µM) depolymerizing drugs for 30 min at 37°C. Lysosome distribution percentage was calculated (n=30 cells). (b) HeLa cells were transfected to silencing indicated dynein chains for 72 h and percentage of cells displaying the indicated phonotype was calculated (n=100 cells). Data are mean ± sem. Scale bar 10 µm. **p<0.01***; p<0.001 (Student's t-test).

a

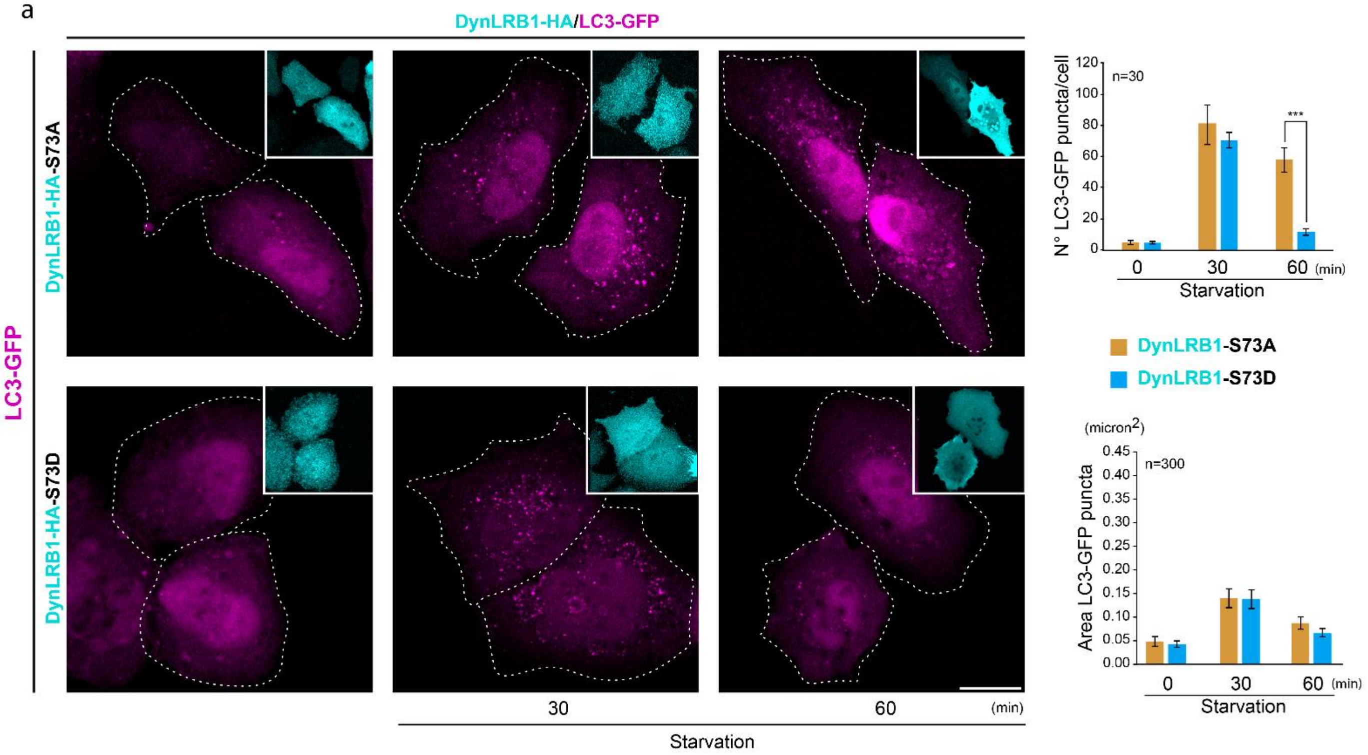

Supplementary Figure 3. DynLRB1-S73A impaired LC3-autophagy flux.

HeLa cell were transfected to co-express separately HA-tagged version of DynLRB1-S73A and S73E with LC3-GFP. 48 h post-transfection the cells were starved on HBBS buffer for 30-60 minutes and then immunofluorescence against HA was performed. Representative images of time 0, 30 and 60 min are shown. The number (n=30 cells) and size of LC3-positive puncta (n=300 puncta) were measured. Scale bar 10 µm. **p<0.01***; p<0.001 (Student's t-test).

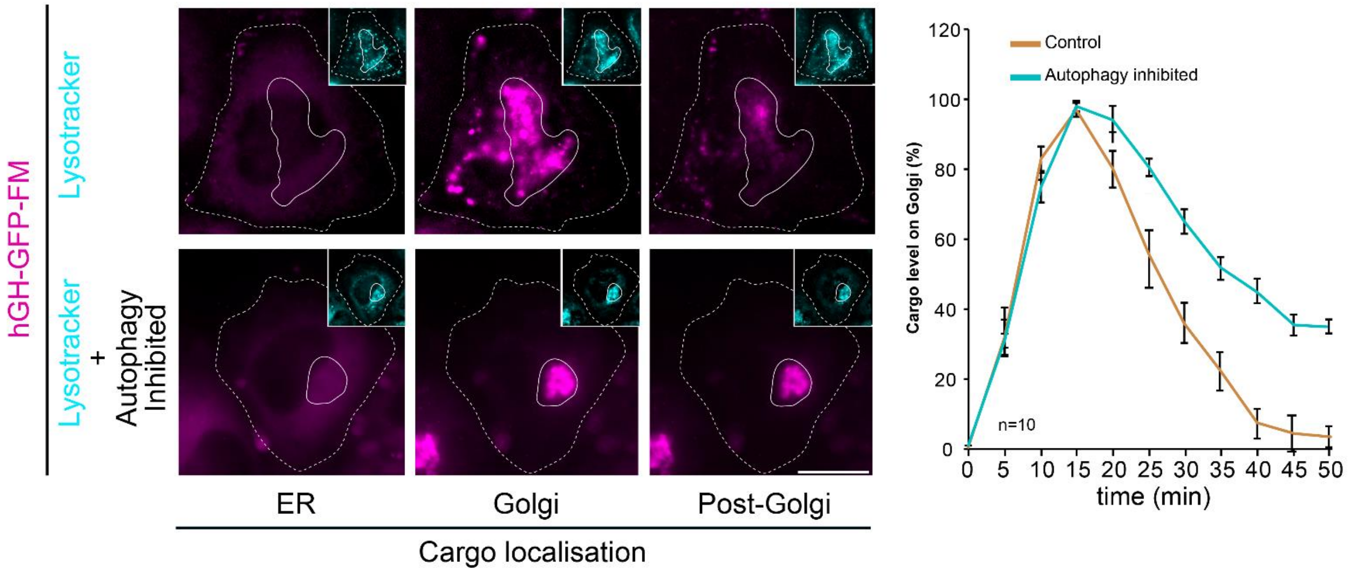

Supplementary Figure 4. Pharmacological inhibition of macroautophagy reduces cargo secretion.

HeLa-hGH-GFP-FM cells were subjected to the traffic assay in the absence or presence of VPS34 inhibitor (1 µM SAR405). The subcellular distributions of cargo and lysosomes were recorded by live-cell imaging (n=10 cells). Data are means ±SEM. Scale bar, 10 µm. **, p <0.01; ***, p <0.001 (Student's t-tests).

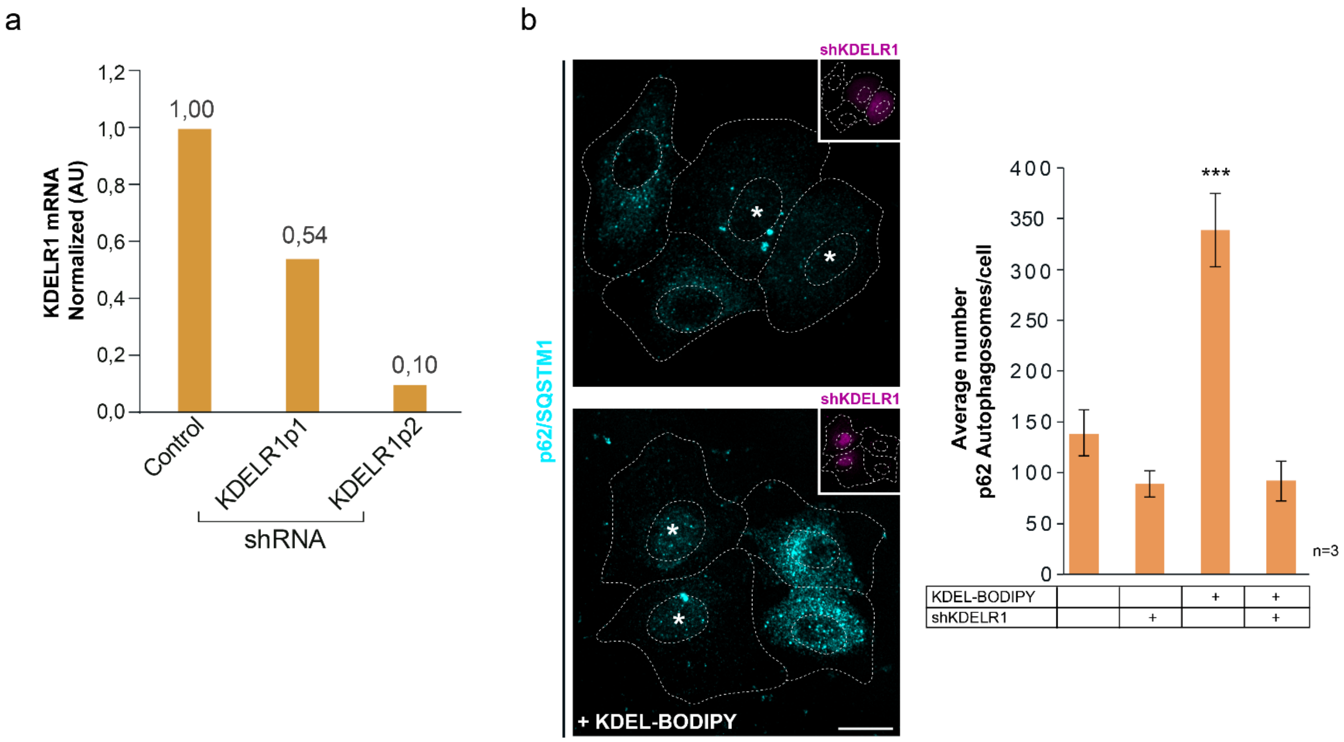

Supplementary Figure 5. KDELR 1 is needed to increase p62/SQSTM1 positive autophagosomes.

(a) KDELR1 was silenced on HeLa cells by using KDELR1p1 or KDELR1p2 shRNAs. After 48 h the cells were solubilized, and mRNA was extracted to perform qPCR. shRNA KDELR1p2 was more efficient to reduce KDELR1 mRNA level (n=2 independent experiments). (b) HeLa cells were transfected as in (a) with KDELR1p2 shRNA. After 48 the cells were incubated with KDEL-BODIPY peptide (1 µM) for 15 minutes at 30°C, then fixed and immuno- stained against p62/SQSMT1.The number of p62 autophagosomes were quantified (n=3 independent experiments) Scale bar 10 µm. **p<0.01***; p<0.001 (Student's t-test).

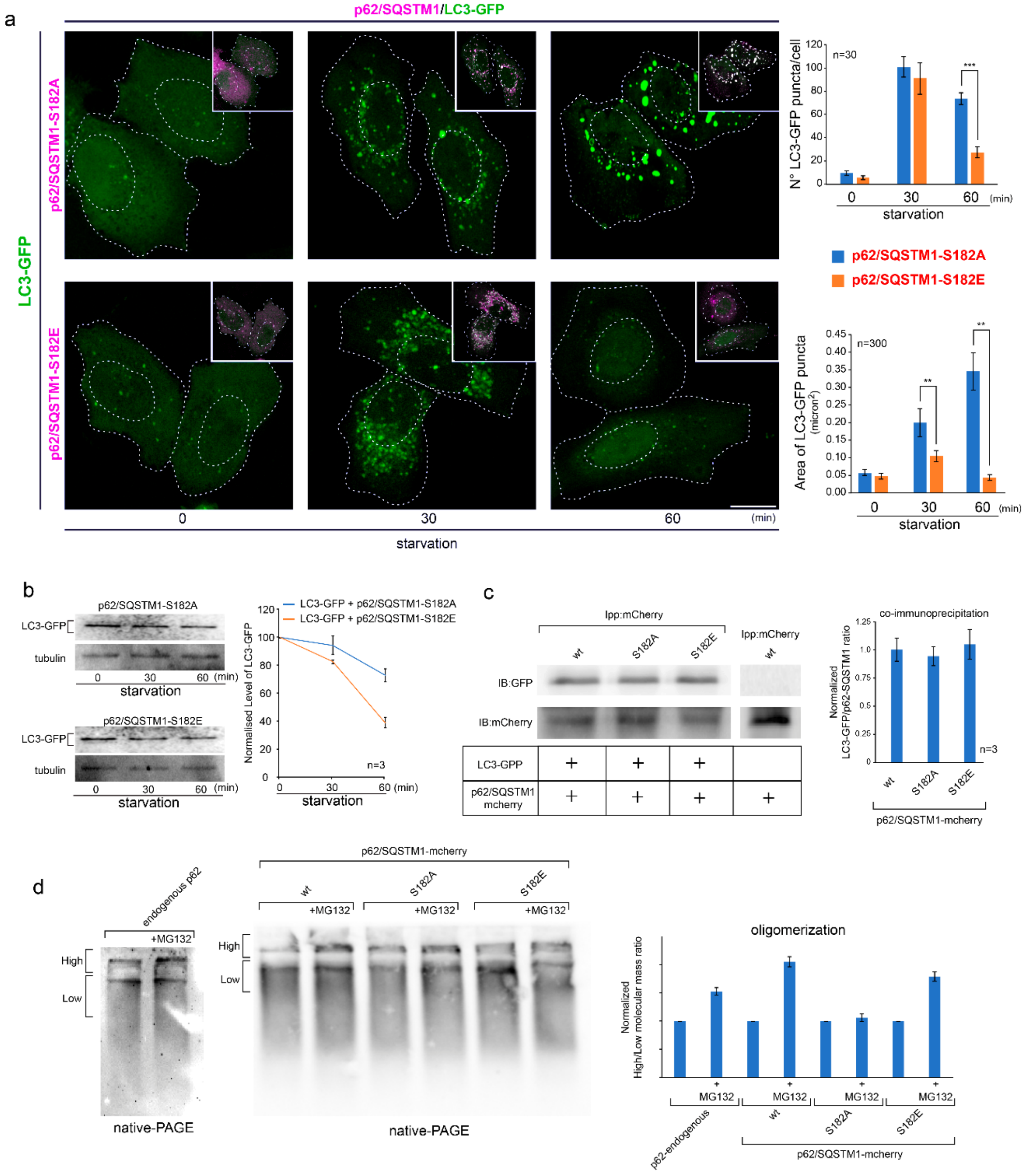

Supplementary Figure 6. p62/SQSTM1-S182A altered autophagy flux by impaired oligomerization.

(a) HeLa cells were transfected to co-express LC3-GFP and p62/SQSTM1-S182A or p62/SQSTM1-S182E. 48 h post-transfection the cells were starved on HBBS buffer for 30-60 min and then fixed. Representative images of time 0, 30 and 60 min are shown. The number (n=30 cells) and size of LC3-positive puncta (n=300 puncta) were measured. (b) HeLa cells were transfected to co-express mcherry-tagged versions of p62/SQSTM1-S182A or p62/SQSTM1-S182E with LC3-GFP. 48 h post-transfection the cells were starved on HBBS buffer for 60 min. Representative images of time 0, 30 and 60 min are shown (n=3 independent experiments). (c) HeLa cells were transfected to co-express mcherry-tagged versions of p62/SQSTM1-wt, S182A and S182E with LC3-GFP. 48 h post-transfection the cells were lysed, and immunoprecipitation was performed with polyclonal antibody against mcherry using magnetic DynaBeads. Samples were separated by SDS-PAGE and immunoblot was performed with polyclonal anti-GFP antibody to detect LC3-GFP co-immunoprecipitation. LC3-GFP:p62-SQSTM1 ratio was calculated and normalized to wt (S182S). (n=3 independent experiments). (d) HeLa cell were transfected to express mcherry-tagged version of p62/SQSTM1-wt, S182A and S182E. 48 h post-transfection the cells were treated or not with the proteasomal inhibitor MG132 (1 µM) by 16 h to induce p62/SQSTM1 oligomerization. The cells were then lysed, and the samples were separated by native-PAGE and immunoblot was performed with polyclonal anti mcherry antibody. High/low molecular mass p62/SQSTM1 ratio was calculated and is shown as normalized ratio (n=3 independent experiments). Data are means ±SEM. Scale bar, 10 µm. **, p<0.005; ***, p <0.001 (Student's t-tests).

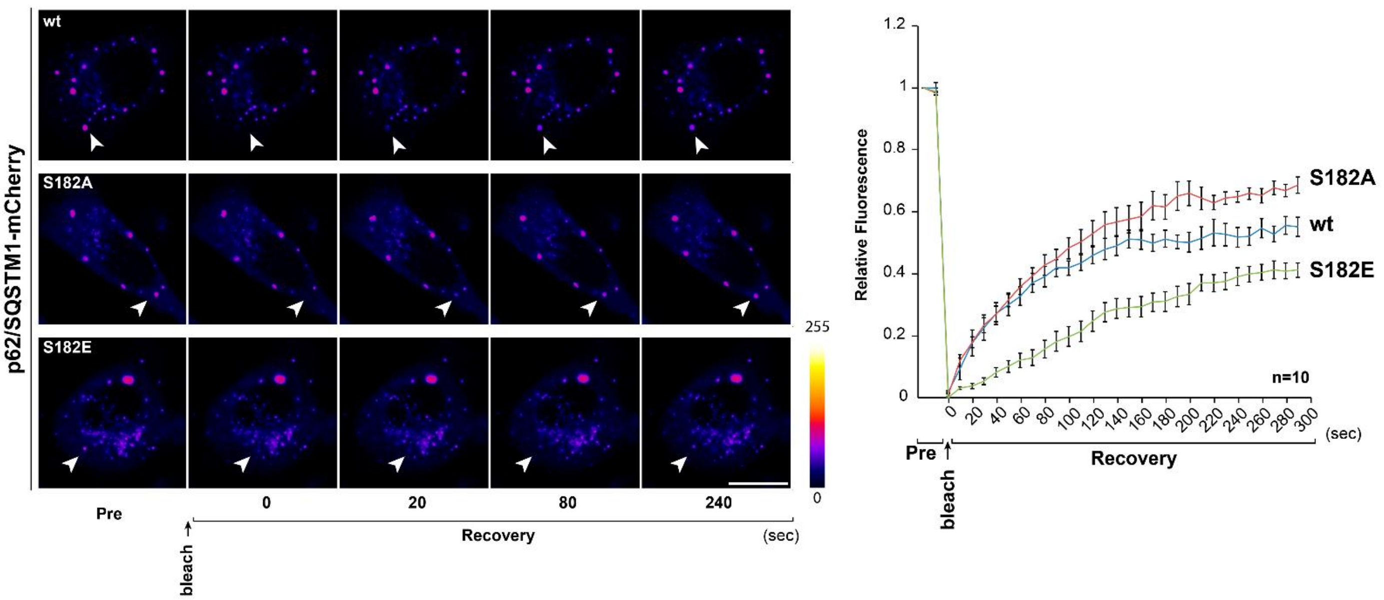

Supplementary Figure 7. p62/SQSTM1-S182A and p62/SQSTM1 have distinct dynamic.

HeLa cells were transfected to mcherry-tagged version of p62/SQSTM1-wt, S182A and S182E. After 48 h the cells were placed on an Okolab temperature control chamber at 37°C on phenol red free DMEM-HEPES, 5% FBS. FRAP analysis was performed using the FRAP wizard module and the LASX software. Scale bar 10 µm. The fluorescence recovery in a p62/SQSTM1 body was measured and the relative fluorescence was determined at each time point and is shown as FireLUT. Recovery is represented as the mean ± SEM (n=10 cells). Scale bar 10 µm.

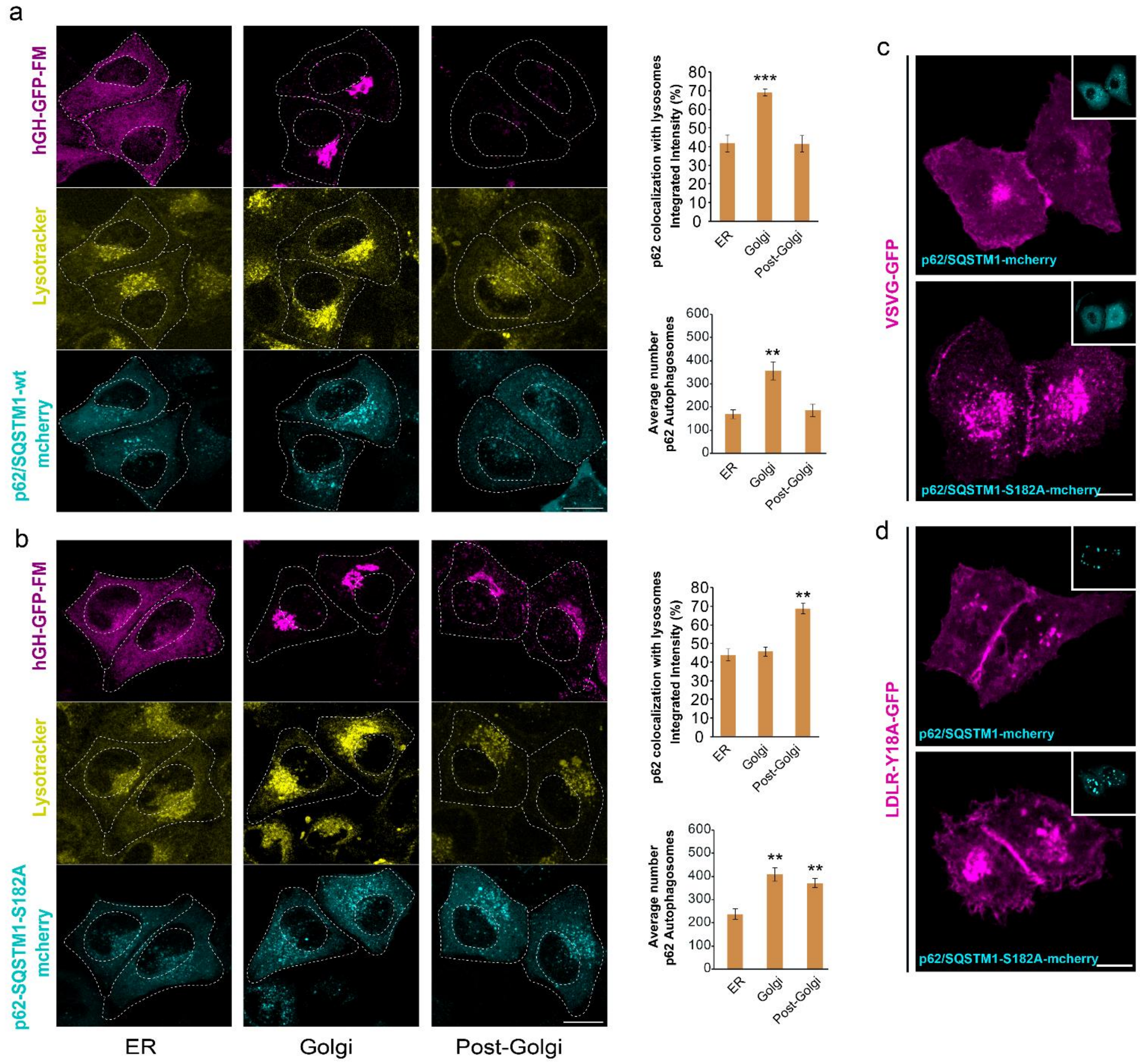

Supplementary Figure 8. Functional p62/SQSTM1 is required to sustain Golgi to PM transport.

HeLa-hGH-GFP-FM were transfected to express p62-mcherry (a) or p62-S182A mutant (b) and incubated with DeepRed-lysotracker. Then the cells were subjected to a transport assay. Number and colocalisation of p62/SQSTM1 was assessed by quantitative image analysis (n=30 cells). (c) HeLa cells were transfected separately to express the transmembrane proteins VSVG and LDLR-Y18A (endocytosis defective mutant) in combination with p62-mcherry or p62-S182A mutant. Then the cells were fixed and localisation on cell surface or Golgi was imaged. (n= 30 cells). Scale bar 10 µm. **p<0.01***; p<0.001 (Student's t-test).

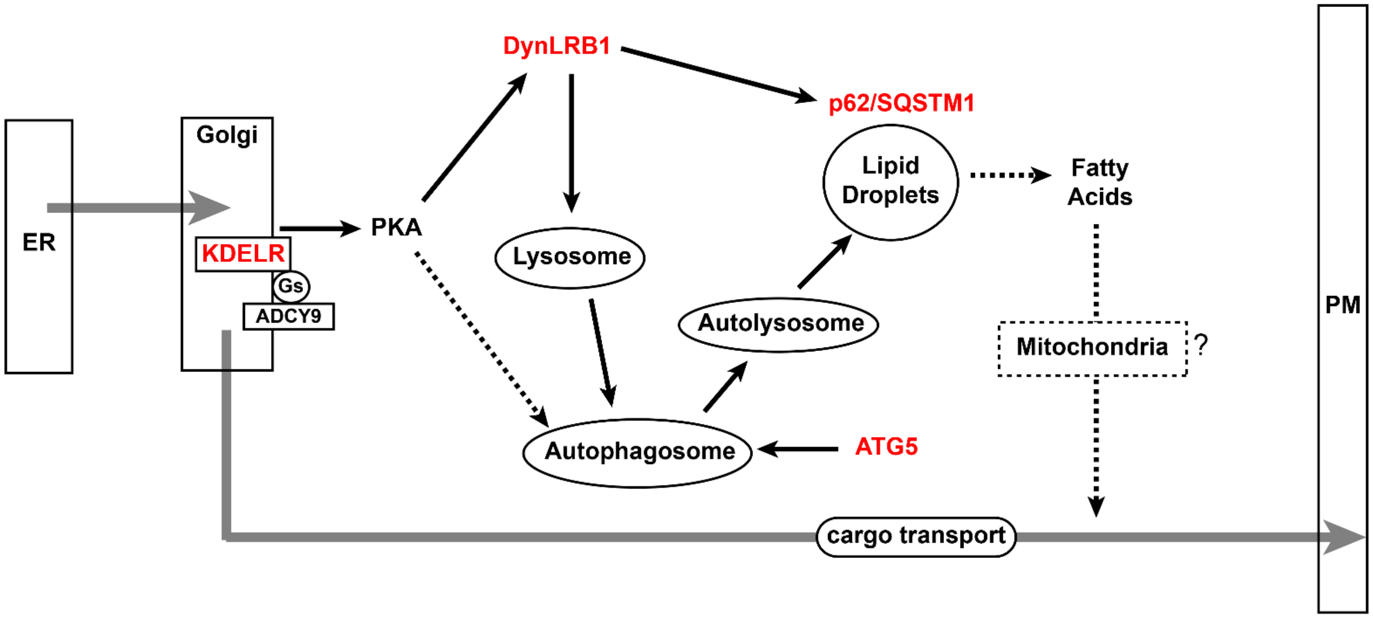

Supplementary Figure 9. Inter-organelle signaling crosstalk regulates membrane transport, by regulate autophagy and lipid droplet turnover.

During ER to Golgi membrane transport, a KDELR-dependent signaling trigger a lysosome repositioning and autophagosome formation regulated by DynLRB1 and p62/SQSTM1, respectively. This Golgi-orchestrated response of lysosomes and autophagosomes has and revealed an uncharacterized physiological role of lysosomes and autophagy as positive regulators of membrane transport. Disturbing lysosome repositioning or autophagosome formation by using phospho-defective DynLRB1 or p62/SQSTM1 mutants impacts strongly on secretion from Golgi. Both, DYNLRB1 and p62/SQSTM1 regulates lipid droplets turnover. ATG5 and p62/SQSTM1 regulates lipid droplet turnover by regulation formation and degradation, respectively. Reduction on the number and content of lipid droplet reduces cargo secretion from Golgi.
