# Supplementary material for: KDEL receptor regulates secretion by lysosome relocation- and autophagy-dependent modulation of lipid-droplet turnover
Source: Nat Commun. 2019 Feb 13;10:735. doi: 10.1038/s41467-019-08501-w (PMC6374470; doi:10.1038/s41467-019-08501-w)
Supplement: Supplementary file 8 — Description of Additional Supplementary Files [file 41467_2019_8501_MOESM8_ESM.docx]

Description of Additional Supplementary Files

**Supplementary Movie S1.** p62/SQSTM1 interacts dynamically with LDs. H4 cells were transfected to transiently express the p62/SQSTM1-wt (mcherry) and the LDs were stained by incubation with BODIPY™ 493/503. Cells on MatTek glass-bottom dishes were incubated on red-Phenol free DMEM-Hepes 10 mM, pH7.4 at 37°C and time lapse images were acquired using a TCS SP8 Leica confocal microscope.

**Supplementary Movie S2.** Phospho-mimetic p62/SQSTM1 increases interaction with LDs. H4 cells were transfected to transiently express the p62/SQSTM1-S182E (mcherry) and the LDs were stained by incubation with BODIPY™ 493/503. Cells on MatTek glass-bottom dishes were incubated on red-Phenol free DMEM-Hepes 10 mM, pH7.4 at 37°C and time lapse images were acquired using a TCS SP8 Leica confocal microscope.

**Supplementary Movie S3.** Phospho-inert p62/SQSTM1 do not interacts with LDs. H4 cells were transfected to transiently express the p62/SQSTM1-S182A (mcherry) and the LDs were stained by incubation with BODIPY™ 493/503. Cells on MatTek glass-bottom dishes were incubated on red-Phenol free DMEM-Hepes 10 mM, pH7.4 at 37°C and time lapse images were acquired using a TCS SP8 Leica confocal microscope.

**Supplementary Movie S4.** Phospho-inert DynLRB1 reduces p62/SQSTM1 interaction with LDs. H4 cells were transfected to transiently express the DynLRB1-S73A and p62/SQSTM1-wt (mcherry) and the LDs were stained by incubation with BODIPY™ 493/503. Cells on MatTek glass-bottom dishes were incubated on red-Phenol free DMEM-Hepes 10 mM, pH7.4 at 37°C and time lapse images were acquired using a TCS SP8 Leica confocal microscope.

**Supplementary Movie S5.** Phospho-mimetic DynLRB1 increases p62/SQSTM1 interaction with LDs. H4 cells were transfected to transiently express the DynLRB1-S73D and p62/SQSTM1-wt (mcherry) and the LDs were stained by incubation with BODIPY™ 493/503. Cells on MatTek glass-bottom dishes were incubated on red-Phenol free DMEM-Hepes 10 mM, pH7.4 at 37°C and time lapse images were acquired using a TCS SP8 Leica confocal microscope.
